# Clinical use of CCR5 inhibitors in HIV and beyond

**DOI:** 10.1186/1479-5876-9-S1-S9

**Published:** 2011-01-27

**Authors:** Bruce L Gilliam, David J Riedel, Robert R Redfield

**Affiliations:** 1 Institute of Human Virology, University of Maryland School of Medicine, 725 West Lombard St., Baltimore, 21201 Maryland, USA

## Abstract

Since the discovery of CCR5 as a coreceptor for HIV entry, there has been interest in blockade of the receptor for treatment and prevention of HIV infection. Although several CCR5 antagonists have been evaluated in clinical trials, only maraviroc has been approved for clinical use in the treatment of HIV-infected patients. The efficacy, safety and resistance profile of CCR5 antagonists with a focus on maraviroc are reviewed here along with their usage in special and emerging clinical situations. Despite being approved for use since 2007, the optimal use of maraviroc has yet to be well-defined in HIV and potentially in other diseases. Maraviroc and other CCR5 antagonists have the potential for use in a variety of other clinical situations such as the prevention of HIV transmission, intensification of HIV treatment and prevention of rejection in organ transplantation. The use of CCR5 antagonists may be potentiated by other agents such as rapamycin which downregulate CCR5 receptors thus decreasing CCR5 density. There may even be a role for their use in combination with other entry inhibitors. However, clinical use of CCR5 antagonists may have negative consequences in diseases such as West Nile and Tick-borne encephalitis virus infections. In summary, CCR5 antagonists have great therapeutic potential in the treatment and prevention of HIV as well as future use in novel situations such as organ transplantation. Their optimal use either alone or in combination with other agents will be defined by further investigation.

## Introduction

After the discovery that HIV gains entry to cells by binding the CD4 receptor [1], research initially focused on development of inhibitors that could block this binding step. However, this line of inquiry led to the realization that CD4 receptor binding was necessary but not sufficient for HIV to enter the host cell; a second step – a coreceptor – was also required. The coreceptors CCR5 (CC chemokine receptor 5) [2-5] and CXCR4 (CXC chemokine receptor 4) [6-9] were discovered a few years later. Identification of the three natural ligands of CCR5 – (*R*egulated upon *A*ctivation, *N*ormal *T*-cell *E*xpressed, and *S*ecreted [RANTES], macrophage inflammatory protein-1 alpha [MIP-1α], and macrophage inflammatory protein-1 beta [MIP-1β]) – as potent inhibitors of HIV [10] quickly led to research to find synthetic compounds to block the receptor and thus prohibit viral entry.

CCR5 is expressed on a number of cells including activated T lymphocytes, macrophages, and dendritic cells [11], and CCR5-tropic HIV-1 strains are predominantly involved in transmission of the virus [12]. Mutation in the CCR5 gene leading to a 32-base pair deletion (∆32) in the CCR5 protein and an absence of CCR5 on the surface of cells causes homozygotes for ∆32 to be almost completely resistant to HIV-1 infection [13-15]. Additionally, heterozygotes for ∆32 have delayed HIV disease progression, slower declines in CD4 cell counts, and lower average circulating viral loads [16]. Taken together, this information provided an enticing target for pharmaceutical intervention against HIV-1 infection.

Several products targeted against CCR5 have been developed (Table 1), though only one is currently approved by the U.S. Food and Drug Administration (FDA) for treatment of HIV-1 infection. The pharmaceutical agents active against CCR5 represent a completely new class of antiretroviral drugs, ones that are not antiretrovirals in the strict sense that they are not directed against HIV’s various enzymes, but instead block a host cell receptor to diminish HIV’s access to the host’s cells. Five different CCR5 antagonists – maraviroc, vicriviroc, aplaviroc, INCB009471 and TBR 652 – have been developed and brought to human trials (Table 1). Aplaviroc advancement was halted after results of two Phase IIb trials showed higher than expected rates of idiosyncratic hepatotoxicity [17]. Vicriviroc is currently in advanced clinical development (Phase III) but has yet to be FDA-approved. Maraviroc was initially approved by the FDA in August 2007 for the treatment of HIV-infected patients experiencing virologic failure due to resistance to other classes of antiretroviral drugs and, subsequently, for the treatment of antiretroviral naïve patients. INCB009471 is an oral CCR5 antagonist with an extended half-life such that it can be given once daily; results from a Phase I and II trials were presented in 2007 [18-20], but the company has decided not to continue further trials. TBR 652 is the fifth new CCR5 antagonist, and Phase I trial results were presented at a conference in early 2010 [21,22]. The novelty of this class of therapeutics is that this is the first class of anti-HIV drugs that focus exclusively on host cellular pathways and not on direct inhibition of viral enzyme mechanisms.

**Table 1:** CCR5 Antagonists in Clinical Trials

Product	Mechanism of Action	Company	Status
**Maraviroc**	Non-competitive inhibitor	Pfizer	FDA Approved
**Vicriviroc**	Non-competitive inhibitor	Schering-Plough	Phase III completed
**Aplaviroc**	Non-competitive inhibitor	GlaxoSmithKline	Development discontinued
**INCB009471**	Non-competitive inhibitor	Incyte	Phase I/IIa completed
**TBR 652**	Non-competitive inhibitor	Tobira	Phase II completed
**Pro 140**	Antibody	Progenics	Phase II
**HGS004**	Antibody	Human Genome Sciences	Phase I completed

Two other products in development are antibodies to the CCR5 receptor: PRO 140 and HGS004. PRO 140 is a humanized monoclonal antibody that binds to CCR5 and inhibits CCR5-tropic HIV-1 *in vitro*, and it was recently shown to have potent antiviral activity after a single dose in a Phase Ib monotherapy, dose escalation trial [23]. HGS004 is a human immunoglobulin G4 monoclonal antibody against CCR5 that was also recently tested in a Phase Ib trial [24] that established its safety and *in vivo* activity against HIV-1.

## Mechanism of action

The process by which HIV infects a host cell is complicated and requires multiple steps. First, the env protein (gp120) on the surface of the virus binds to cellular CD4 receptors. The binding of gp120 leads to a conformational change that exposes the V3 loop; the exposed V3 loop of gp120 then interacts with and binds to a coreceptor on the host cell (either CCR5 or CXCR4) [25]. After the coreceptor is bound, another conformational change in the viral envelope unmasks gp41, which can then insert into the cell’s membrane [26]. This step brings the virus into close proximity with the cell, leading to fusion of the virus with the cell [26]. CCR5 antagonists bind to the CCR5 receptor and induce a conformational change to it such that the V3 loop of the viral gp120 is unable to recognize and bind [27-30]. CCR5 antagonists act as allosteric, non-competitive inhibitors of the receptor [25]. CCR5 antibodies work by binding to the extracellular domain of the CCR5 receptor and thereby inhibit interaction between gp120 and the coreceptor [31,32]. The result of binding of either an antagonist or an antibody is blockade of the binding interaction which prevents HIV from entering the host cell.

## Tropism

As noted above, the structural change that occurs after CD4 binding leads to exposure of the V3 loop of gp120, and this V3 loop is the area of the envelope that interacts with the coreceptor. The amino acid sequence of the V3 variable domain appears to be the primary determinant of which coreceptor is utilized, i.e. the tropism of the virus [33]. Tropism refers specifically to which coreceptor the virus is designed to utilize to gain entry to host cells. There are 4 categories of HIV-1 tropism: 1) R5 – viruses that bind only to the CCR5 coreceptor; 2) X4 – viruses that bind only to the CXCR4 coreceptor; 3) dual tropism – viruses that can bind to either coreceptor; and 4) mixed tropism – mixed populations that include both R5- and X4-tropic viruses [34,35].

An important relationship between tropism/coreceptor usage and different phenotypic characteristics of the virus has been clearly established. Originally, *in vitro* studies demonstrated that viruses that were syncytium-inducing on T-cell lines and preferentially replicated on T lymphocytes were more pathogenic [36]; these features were also correlated with more rapid progression to AIDS and AIDS-related mortality [37,38] and were eventually identified as X4-tropic viruses. Non-syncytium-inducing viruses were noted to replicate best in monocyte-macrophages, have a less virulent clinical course, and correspond to R5-tropic viruses [36-40]. The dynamic nature of HIV tropism has important ramifications for viral transmission and pathogenicity.

R5-tropic viruses predominate in transmission events and infection of new patients as they appear to be more efficiently transmitted than X4-tropic strains [41]; additionally, R5-tropic viruses are the principal circulating strain in most patients with early HIV infection [42]. Further evidence, albeit indirect, for the predominance of R5-tropic virus in transmission events comes from the relative resistance to infection of homozygotes for ∆32 within the CCR5 gene [13-15]. One factor that is likely to play a role in the preferential transmission of R5-tropic strains is the high levels of expression of CCR5 on cells in the genital mucosa [43], thus allowing R5-tropic strains an easier pathway into the host.

X4-tropic viruses are found much less often in the early stages of HIV infection and are thought to be uncommonly involved with transmission events. However, there is a strong correlation between disease progression to AIDS and coreceptor switching from CCR5 to CXCR4 [40]. It is not entirely clear if this association is causal, i.e. whether CXCR4 receptor usage is pathogenic in leading to clinical progression to AIDS, or if it is simply a consequence of disease progression [44]. Several studies have shown a significantly increased risk of disease progression among patients with X4-tropic or dual/mixed virus [42,45,46]. One of the main (yet still theoretical) concerns about the clinical use of CCR5 antagonists is that they might promote the emergence of X4-tropic viruses that could then go on to accelerate disease progression to AIDS [44]. At this point in time, however, accumulating clinical trial data has not substantiated this fear.

The majority of reports on tropism have come from cohorts of patients infected with HIV-1 subtype B (from the U.S. and western Europe), but new research is emerging to suggest that tropism is also affected by HIV-1 subtype or clade [47]. X4- and mixed tropic viruses appear to be much less common in subtype C-infected patients, and this relationship appears to hold regardless of CD4 cell count or disease stage [48-50]. In subtype C-infected patients, X4-tropic strains have been found after prolonged treatment with ART. Differences in tropism between subtypes B and C could be related to intrinsic differences in the conformation of the V3 loop that could affect the evolution of HIV from R5- to X4-tropic [51]. The prevalence of X4-tropic virus also appears to be lower in subtype A [52], but another study found X4-tropic viruses emerging in patients with progression to AIDS [53]. Two small studies with subtype D viruses have found a higher proportion of X4- and mixed-tropic viruses [54,55], whereas two small studies of subtype E viruses (more prevalent in southeast Asia) found a similar proportion of R5-, X4-, and mixed tropic viruses as that usually found with subtype B [56,57]. Overall, the different subtypes appear to have considerable variability in coreceptor usage, and these differences may have a significant impact on the ability to use CCR5 antagonists in different countries.

From a clinical standpoint, it is imperative to assess viral tropism prior to prescribing a CCR5 antagonist since the drugs are only effective against R5-tropic viruses. Tropism can be evaluated by genotypic or phenotypic assays. Genotypic assays evaluate the amino acid sequence of the V3 region of gp120, the primary determinant of tropism [58]. Genotypic algorithms to predict viral tropism based on V3 genetic sequences are in development [59], though the results have not always correlated well with phenotypic assays [58]. At this time, genotypic assays have not yet entered into clinical practice.

Phenotypic assays are the most widely used to date in clinical practice; the original test was the Trofile™ assay by Monogram Biosciences [60]. This test identified X4-tropic strains with a sensitivity of 10%, but it could not differentiate between dual-tropic viruses and mixed populations of X4- and R5-tropic strains [58,61]. The Trofile™ test had the disadvantages of being expensive and both time- and labor-intensive. Additionally, the fact that not all minority variants are detected with this assay is suboptimal because these strains can lead to treatment failure if undetected.

Failure of the Trofile™ assay to detect minority variants led to the development of an enhanced sensitivity tropism assay. This assay is able to detect X4 virus with a sensitivity of 100% when at least 0.3% of the viral population is X4-tropic [25]. Using this enhanced assay on previously collected samples from major CCR5 antagonist trials found that a large number of virological failures were explained by pre-existing X4-tropic virus that was not detected using the original Trofile™ assay [62,63]. These results reinforce the importance of these assays, though further advances will be necessary before they become standard in routine clinical practice.

## Pharmacology

Maraviroc is administered orally at a usual dose of 300 mg twice daily and can be given without regard to fasting or fed state. It is rapidly absorbed and has peak drug concentrations between 30 minutes and 4 hours after dosing [64]. At the usual dose of 300 mg, bioavailability is 33% [65], and steady state is reached within 7 days [64]. The drug is widely distributed in the body [65]; preclinical studies with rats found poor penetration into the central nervous system [66], but several small studies in humans demonstrated that maraviroc enters the cerebrospinal fluid at therapeutic levels [67-69]. Tiraboschi et al [67] also found maraviroc levels several times the IC50 in semen. Another study examined the concentration of maraviroc in cervicovaginal fluid and found that drug levels were significantly higher than in plasma at the same time and even 72 hours after oral dosing [70]. This result could have important implications for prevention of transmission [see Potential uses below].

Maraviroc is a substrate for the hepatic cytochrome P450 enzyme, CYP3A4 (though it does not inhibit or induce the enzyme itself), as well as P-glycoprotein [65], and so dosing adjustments are required when it is given in combination with other inducers and inhibitors of these enzymes. For inhibitors of CYP3A4 like protease inhibitors, itraconazole, or clarithromycin, the dose of maraviroc is reduced to 150 mg twice daily [65,71]. With inducers of CYP3A4 (e.g. efavirenz or rifampin), maraviroc dosing should be increased to 600 mg twice daily [65,72]. Dosing does not need to be adjusted when used in combination with other antiretrovirals like tenofovir, nevirapine, enfuvirtide, or raltegravir. Maraviroc concentrations were mildly increased in HIV negative patients with mild and moderate hepatic impairment, though no change in dosing was recommended [65,73]. The majority of the drug is excreted unchanged in feces (~75%) while about 20% is excreted in urine [64]. No dosage adjustment is necessary in patients with renal insufficiency. Lastly, maraviroc pharmacokinetics were not found to be altered in men vs. women or in patients of various races/ethnicities [65].

## Clinical experience with CCR5 antagonists

To date there has only been one CCR5 antagonist, maraviroc, approved for the treatment of HIV by the U.S. FDA. This review will therefore focus on maraviroc except where other agents in development have unique characteristics or findings that are important to understand when considering the CCR5 antagonist class.

### Efficacy

The clinical experience with maraviroc (MVC) in HIV infected patients essentially began with two small phase IIa dose escalation studies evaluating the antiviral efficacy of MVC monotherapy over a period of 10 days [74]. These studies evaluated MVC at multiple doses in fed and fasted states compared to placebo in 82 patients. Study participants had to have CCR5 tropic virus, and the mean baseline CD4 cell count and viral load was 544 cells/mm^3^ and 4.62 log_10_ HIV-1 RNA copies/ml, respectively. Of the 63 patients who completed 10 days of therapy, all who received MVC at least 100 mg once or twice daily achieved a mean reduction of viral load at Day 11 of 1.13-1.60 log_10_ HIV-1 RNA copies/ml. The lower dose groups achieved reductions of 0.43 and 0.66 log_10_ HIV-1 RNA copies/ml. Interestingly, the maximal viral load decline occurred in several patients after discontinuation of the drug. The median time to viral load nadir was 10-15 days in the different dose groups and viral rebound did not occur immediately.

### Treatment experienced patients

Following the viral load reductions seen in the Phase II studies, maraviroc was evaluated in R5-tropic, treatment-experienced HIV-infected patients [75]. Patients were randomized to receive once or twice daily MVC in combination with an optimized background regimen (OBR) vs. an OBR (the placebo arm) alone in the MOTIVATE (*M*araviroc versus *O*ptimized *T*herapy *i*n *V*iremic *A*ntiretroviral *T*reatment-*E*xperienced Patients) trials. These trials were conducted in Canada and the U.S. (MOTIVATE 1) and Australia, Europe and the U.S. (MOTIVATE 2). The results of the trials were pooled for analysis with a total of 1,049 patients (414 MVC once daily; 426 MVC twice daily; 201 placebo (OBR)). It is notable that 955 other patients who were screened for these two studies were excluded due to dual, mixed or CXCR4 tropism. The primary study endpoint, the mean change from baseline in the log_10_ HIV RNA levels at 48 weeks, was -1.68 for MVC once daily, -1.84 for MVC twice daily and -0.79 for the placebo arm. More importantly, the percentage of patients achieving a viral load of < 50 copies/ml at 48 weeks was 43% for MVC once daily, 46% for MVC twice daily and 17% for the placebo arm, which were statistically significant between each MVC arm and placebo arm (p < 0.001). There were also statistically significant greater mean CD4 cell count increases from baseline in the MVC arms (116 cells/mm^3^ in MVC once daily, 124 cells/mm^3^ MVC twice daily, 61 cells/mm^3^ in placebo) [76]. Subanalyses of the pooled MOTIVATE results revealed a treatment benefit of MVC in combination with OBR when compared to placebo in combination with OBR at screening viral load < or > 100,000 copies/ml, baseline CD4 cell count at all strata (< 50, 50-100, 101-200, 201-350, and > 350 cells/mm^3^), baseline R5 tropism, viral subtype (B vs. non-B), enfuvirtide use, ∆32 genotype, race, gender, genotypic susceptibility score (GSS), phenotypic susceptibility score, overall susceptibility score, and first use of enfuvirtide, lopinavir-ritonavir or tipranavir-ritonavir [77]. Importantly, these analyses revealed a significant benefit of using an additional active new antiretroviral agent in combination with maraviroc. The results of these studies led to FDA approval of maraviroc for treatment experienced patients.

Those patients who failed screening for the MOTIVATE trials due to dual or mixed tropic virus were offered the opportunity to enroll in a trial evaluating maraviroc in treatment experienced patients with dual, mixed or CXCR4 tropism. In this study 186 patients were randomized to once or twice daily MVC or placebo in combination with an OBR [78], and there were 167 evaluable patients. The primary endpoint, the mean change in viral load from baseline at 24 weeks, was 0.91, 1.20 and 0.97 log_10_ copies/ml HIV RNA in the once daily MVC, twice daily MVC and placebo arms, respectively. Similarly, the proportion of patients with HIV RNA < 50 copies/ml was 21%, and 27%, and 16% (once daily MVC, twice daily MVC, placebo). Neither noninferiority or superiority of the MVC arms in comparison with the placebo arm was established. Mean CD4 cell count increases from baseline were 60 cells/mm^3^ in the once daily MVC arm, 62 cells/mm^3^ in the twice daily MVC arm , and 36 cells/mm^3^ in the placebo arm at 24 weeks. These differences were statistically significant at 24 weeks, but they were no longer statistically significant at 48 weeks. Overall, there was little virologic or immunologic benefit of maraviroc for the treatment of dual or mixed tropic virus.

Maraviroc has also been evaluated in combination with raltegravir and etravirine in a review of 28 treatment experienced patients with R5 tropic virus who were started on this combination through expanded access programs. At 48 weeks, all patients had an HIV RNA < 400 copies/ml and 26/28 (93%) had an HIV RNA < 50 copies/ml [79]. There is one case report of a treatment experienced patient with resistant HIV-2 infection that was successfully treated with raltegravir and maraviroc based therapy [80].

### Treatment naïve patients

The success of maraviroc in the treatment experienced trials and the fact that a CCR5 antagonist would have the greatest potential for effectiveness in populations with predominantly CCR5 tropic virus led to an evaluation of MVC in antiretroviral treatment naïve HIV-infected patients. The MERIT (*M*araviroc versus *E*favi*re*nz in *T*reatment-Naive Patients) trial was a phase IIb/III, double-blind, placebo-controlled trial that evaluated the safety and efficacy of MVC vs. efavirenz in patients with R5 tropic virus from Australia, Europe, North America, South America and South Africa [63]. Similar to the MOTIVATE trials, patients were initially randomized to receive MVC 300 mg once or twice daily or efavirenz 600 mg once daily in combination with co-formulated zidovudine and lamivudine. In contrast to the MOTIVATE trials, only 17% of the patients screened for the MERIT study were excluded for having X4 tropic virus. In total, 895 patients were randomized, but an interim analysis found that patients receiving once daily MVC fell outside of the prespecified thresholds for non-inferiority in comparison to efavirenz. The data and safety monitoring board (DSMB) discontinued the once daily MVC arm. This left 721 evaluable patients in the 48 week analysis. In the primary analysis of viral load response < 400 copies/ml, twice daily MVC was non-inferior to efavirenz. However, in the co-primary endpoint of percentage of patients with viral load < 50 copies/ml (65.3% MVC, 69.3% EFV), the non-inferiority criterion was not met. There were also lower virologic response rates noted with MVC in high baseline viral load patients, Southern hemisphere patients, black patients and those with non-B subtype virus.

Upon blinded retesting of screening specimens with the enhanced Trofile™ assay, 15% of patients had CXCR4 tropic virus. Post hoc analysis of the study with these CXCR4 tropic patients excluded revealed that maraviroc twice daily met the non-inferiority criteria with efavirenz (HIV RNA < 50 copies/ml: 68.5% MVC vs. 68.3% EFV). Additionally, response rates for maraviroc in the subgroup analyses improved after reanalysis, particularly in those with high baseline viral load. Interestingly, response rates for maraviroc were higher in the Northern hemisphere, and response rates for efavirenz were higher in the Southern hemisphere. The authors attributed these differences to higher adverse event discontinuation rates for efavirenz in the Northern hemisphere and higher default rates for patients receiving maraviroc in the Southern hemisphere. The differences noted by the DSMB for the once daily maraviroc arm when compared to efavirenz that led to discontinuation of that arm were no longer outside the noninferiority thresholds in the post hoc reanalysis. In both the primary and the post hoc reanalysis, CD4 cell count increases were 26-30 cells/mm^3^ higher in the maraviroc arm. Subsequent to the reanalysis of the MERIT data, maraviroc was approved for use in treatment naïve patients by the U.S. FDA.

## Resistance

Although CCR5 antagonists target a host cell receptor, virologic failure and resistance can still occur. Escape from or resistance to CCR5 antagonists can occur through two different mechanisms. The first is through selection of minority variants of CXCR4 or dual/mixed tropic virus. The second is through development of mutations in the gp120 V3 loop, elsewhere in gp 120, or in gp41 [81-83]. *In vitro*, multiple mutations in the V3 loop can lead to resistance [82,84,85]. However, no signature mutations or consistent patterns have been noted in the same or different isolates, and the mutations appear to be context dependent [82,84,85]. Also, development of cross-resistance to other CCR5 antagonists may or may not occur [81,84-86]. It is interesting to note that *in vitro* primary R5 viruses in PBMCs exposed to CCR5 antagonists usually maintain R5 tropism even when CXCR4 receptors are abundantly available [81,82,84,87]. It has been proposed that switching to CXCR4 does not occur due to decreased fitness of transitional variants and/or sensitivity to CCR5 antagonists [85,88,89].

In clinical studies, dual, mixed or CXCR4 tropic viruses have been detected in a significant proportion of those experiencing treatment failure while receiving maraviroc. Indeed, in the MOTIVATE studies, 57% (76/133) of MVC-treated patients with R5 tropic HIV at baseline who experienced treatment failure had D/M or X4 viruses detected at the time of failure. This is in contrast to 6/95 (6%) of those who received OBR plus placebo [77]. Similarly, in treatment naïve patients in the MERIT study, 9/29 (31%) MVC treated patients developed X4 virus in comparison to 0/13 efavirenz treated patients [63]. In contrast, phase 3 studies of vicriviroc in treatment experienced patients (VICTOR E3 and 4) detected D/M or X4 viruses in 13% (9/71) of patients experiencing virologic failure [90]. However, it is important to note that in the VICTOR studies, 64% of the subjects had at least three active drugs in their regimen which is in contrast to the maraviroc phase 3 studies in treatment experienced patients where only 37% of patients had at least three active drugs in their regimen. This data is consistent with findings in the earlier phase II vicriviroc studies in treatment experienced patients [91,92]. The results with vicriviroc were also similar in treatment naïve patients where 5/26 (19%) virologic failures on the vicriviroc arm developed D/M or X4 viruses [93]. These studies also did not use the newer more sensitive Trofile™ assay, and the patient population was treatment experienced. In one report evaluating four patients who failed vicriviroc therapy, they were found to have the V3 loop mutations associated with failure in minority populations (0.8-2.8%) of baseline samples [94]. Another report documented two patients who developed X4 variants while failing treatment with maraviroc actually had those variants at baseline [95]. Thus, the emergence of X4 tropic virus in these studies has been demonstrated to be most likely due to the expansion of minority CXCR4 variants present at baseline that were not detected with the Trofile™ assay [85,96]. Upon cessation of CCR5 antagonist therapy, those patients who switched to dual or mixed tropism while on therapy demonstrate reversion back to R5 tropism [95,97,98]. This suggests a fitness cost to dual or mixed tropism *in vivo* which has not been observed in *in vitro* culture [84,86,99]. Additionally, a post hoc analysis of the MOTIVATE studies demonstrated a low incidence of resistance developing to maraviroc in failing patients who had never achieved viral suppression or had virologic rebound [100]. This data, in light of a report [101] demonstrating that poor adherence with maraviroc was not associated with the development of resistance, suggest that maraviroc has a high barrier to resistance.

The development of mutations in the V3 loop, gp 120, and gp 41 is the second mechanism that may present as clinical resistance to CCR5 antagonists. These mutations allow the resistant virus to bind to the cell’s CCR5 receptor that is already bound to maraviroc [84]. This results in a plateau effect of the dose-response curve as increasing drug concentration has no impact due to the ability to utilize the maraviroc-bound receptors. *In vitro*, the mutations at residues 316 and 323 have been selected by maraviroc [84]. *In vivo*, mutation combinations involving residues 11, 18, 10, 20, 21, 22, 25, and 26 have been linked with maraviroc treatment failure [102,103]. In this study, it was noted that some dual tropic clones were responsive to maraviroc *in vivo*. One patient failing treatment with vicriviroc was demonstrated to develop V3 loop mutations which were sufficient to confer resistance [97]. Interestingly, the baseline V3 loop sequences returned once therapy with vicriviroc was discontinued. However, others have questioned the role of these mutations in the V3 loop in the development of resistance to maraviroc [104]. Similarly, an evaluation of the viruses from 323 CCR5 antagonist naïve patients revealed that 7.3% had mutation combinations previously described with maraviroc resistance [105]. It is possible that the presence of these mutations at baseline may facilitate more rapid resistance in a patient who does not achieve rapid viral suppression.

The implications of resistance to CCR5 antagonists with respect to other antiretroviral agents has not been well described; however, one report evaluated the implications *in vitro* and demonstrated that resistance to CCR5 inhibitors may increase the sensitivity of the resistant virus to certain neutralizing antibodies [106]. This finding will need further evaluation *in vivo* prior to the consideration of developing new entry inhibitor treatment sequencing strategies.

## Adverse effects

The utility of an antiretroviral depends on its safety and tolerability, as exemplified when a case of severe hepatic cytolysis was reported in a patient being treated with the investigational CCR5 antagonist, aplaviroc [17]. Review of aplaviroc trials revealed higher than anticipated elevations in ALT and total bilirubin in the aplaviroc arms [17]. No associations were noted between plasma drug concentrations and liver enzyme elevations. The conclusion of the analysis was that it was an idiosyncratic hepatotoxicity and was intrinsic to the molecule not the class of drugs. Further aplaviroc development was halted due to this report.

A Phase II trial of vicriviroc raised concerns for malignancy when 6/90 subjects in the vicriviroc arms developed malignancies compared to only 2/28 in the placebo arm [91]. In the vicriviroc arms, there were four lymphomas (2 Hodgkin and 2 non-Hodgkin), a gastric adenocarcinoma, and an HPV-relate squamous cell carcinoma. There were no lymphomas in the placebo arm. Concern for CCR5 antagonism leading to EBV reactivation led to evaluation of the four lymphoma patients, but EBV reactivation could not be demonstrated [107]. An increased rate of malignancies was not seen in the vicriviroc arms of other studies in both treatment experienced and treatment naïve patients including two Phase III studies with 568 patients treated with vicriviroc [90,92,93].

Despite these initial safety concerns with CCR5 antagonists, maraviroc has had a remarkably clean safety and tolerability profile. The treatment related discontinuation rate due to adverse events was 3% or less in the maraviroc arms of the trials in treatment experienced patients [75,78]. In contrast, in treatment naïve patients, adverse event related treatment discontinuations occurred in 4.2% of those treated with maraviroc compared with 13.6% of those treated with efavirenz [63]. Discontinuations occurred earlier with efavirenz (59% in the first 8 weeks) compared to 40% of those treated with maraviroc. There were no significant differences in the incidence of serious adverse events and deaths between the maraviroc and the placebo arms in either the treatment experienced or the treatment naïve trials. In the MOTIVATE trials, Category C events were not different between arms except for esophageal candidiasis which was more common in the maraviroc arms. Category C events in the MERIT trial were twice as common in the efavirenz arm compared to the maraviroc arm. The incidence of malignancies with maraviroc was not significantly different between arms in treatment experienced patients but twice as many were seen in treatment naïve patients treated with efavirenz vs. maraviroc (Table 2).

**Table 2:** Malignancies in Clinical Trials of Maraviroc

	ART Experienced						ART Naive	
**Malignancy**	**MOTIVATE**			**Non-R5 MVC**			**MERIT**	

	**MVC QD**	**MVC BID**	**OBR**	**MVC QD**	**MVC BID**	**OBR**	**MVC**	**EFV**

Lymphoma	2	2	2	0	0	0	1	3
Kaposi Sarcoma	1	2	3	0	0	0	0	1
Other	0	0	0	1	1	1	2	3

In more than 1,300 patients treated with maraviroc in Phase II and III studies, only one case of potentially life-threatening hepatotoxicity has been reported, and isoniazid and/or cotrimoxazole toxicity were actually implicated as etiologic [63,108]. No significant differences in Grade 3/4 AST and ALT elevations between treatment arms in either the treatment experienced or treatment naïve patients have occurred (~3% for maraviroc and placebo). The effect of maraviroc on lipid profile was more favorable than efavirenz in treatment naïve patients with efavirenz having significantly greater increases in total cholesterol (TC), LDL, and triglycerides [109]. A higher proportion of patients exceeded the National Cholesterol Education Program (NCEP) guidelines for treatment of total cholesterol and LDL, at both week 24 and 48, in the efavirenz group (14.4% for TC, 6.0% LDL) compared to the maraviroc group (2.0% TC, 1.3% LDL). When the 10 year cardiovascular risk was calculated using the Framingham equation, the risk was consistently higher in the efavirenz group than in the maraviroc group. The authors concluded that maraviroc had minimal effects of lipid profiles.

The most commonly reported side effects noted with maraviroc in the registrational trials were headache, dizziness, diarrhea, fatigue, upper respiratory tract infection, cough, abdominal pain, nasopharyngitis, rash, and bronchitis. In the MERIT study, diarrhea, vomiting, dizziness, abnormal dreams, cough and rash were more common in the efavirenz arm while bronchitis and nasopharyngitis were more common in the maraviroc arm. Although postural hypotension was noted with high doses of maraviroc early in its development, it was not seen at significantly different rates than the comparator arms in the Phase II/III trials (5% MVC vs. 4% placebo). Similarly, clinically significant QT prolongation was not noted in the Phase IIb/III trials. As noted above the overall rate of adverse events leading to treatment discontinuation were low in all of these studies reinforcing the favorable tolerability profile of this drug.

## Trends in usage, 2010

### Metabolic and cardiovascular benefits

Untreated HIV infection leads to significant cardiovascular morbidity and mortality, and new data has led to recommendations for earlier antiretroviral treatment [110]. As HIV-infected patients live longer and take antiretrovirals for many years, metabolic effects from these agents are of greater concern. A number of agents have been implicated, although the data has been conflicting for agents such as abacavir [111-113]. Antiretroviral agents with more favorable metabolic profiles are clearly needed.

In treatment experienced patients who have a higher rate of cardiovascular disease at baseline, the rate of cardiovascular events seen with maraviroc treated patients was comparable to that reported in cohort studies of treatment experienced HIV patients, though it was higher than in the placebo arms [96]. All of the patients who had cardiac events in the trials had several pre-existing risk factors for cardiovascular disease including diabetes, hypertension, previous myocardial infarction, known coronary artery disease, hyperlipidemia, and smoking. Treatment-naïve patients treated with maraviroc demonstrated a favorable lipid profile and decreased cardiovascular risk when compared to efavirenz. In the context of aging HIV-infected patients, the relatively neutral metabolic profile of maraviroc to date may prove useful in sparing future toxicities with their associated morbidity and mortality.

Patients initiating antiretroviral treatment who have pre-existing metabolic abnormalities may be candidates for use of a CCR5 antagonist such as maraviroc. However, in patients who are virally suppressed but experiencing metabolic complications on their current regimen, tropism testing cannot be done with the currently available phenotypic assay. Other genotypic assays or assays that can be performed on stored specimens are under study but are not currently available. This leaves the clinician in this situation with three options: 1) avoiding the switch to maraviroc due to lack of the tropism test, 2) testing for tropism at baseline prior to initiation of antiretroviral therapy, or 3) using maraviroc without tropism testing. The use of tropism testing prior to initiation of HAART is appealing and may turn out to have some prognostic value but is limited currently by the cost of the assay. If maraviroc is used without testing, it would be reasonable to check a viral load shortly (4-8 weeks) after switching therapy to ensure that viral suppression is maintained.

Development of newer CCR5 antagonists may also have a role in cardiovascular disease. One recent report demonstrated a new CCR5 antagonist that also has CCR2 blocking activity [22]. As CCR2 has been implicated in atherosclerosis, this could prove to be beneficial [114].

### Tuberculosis

Management of the HIV-infected patient with tuberculosis is challenging due to drug-drug interactions between rifampin and antiretrovirals with similar metabolism through the hepatic cytochrome P450 system. This situation is more difficult in the setting of treatment experienced patients with limited treatment options. Although maraviroc is a substrate for CYP3A4, it can be dosed with rifampin by doubling the dose of maraviroc to 600 mg twice daily [65].

### Hepatitis B and C coinfection

Maraviroc did not appear to have a significant influence on the incidence of hepatic adverse events in patients coinfected with hepatitis B or C, though this assessment was based on very small numbers of coinfected patients [77]. Though maraviroc has proved safe to date in the small number of coinfected patients treated, CCR5 antagonism may have potential implications for the modulation of hepatitis B and C infections, independent of its effect on HIV. CCR5 has been implicated in the recruitment of T cells to the liver in chronic viral hepatitis leading to increases in inflammation [115]. One study in 283 women with hepatitis C suggested less severe hepatic inflammatory scores in individuals heterozygous for CCR5∆32 [116]. However, an analysis of 14 studies could find no association between susceptibility to hepatitis C and CCR5∆32, but the lack of consistency between studies prevented an evaluation of the impact on liver fibrosis [117]. In contrast, the absence of CCR5 has been associated with recovery from hepatitis B [118,119]. Administration of anti-CCR5 monoclonal antibodies has decreased liver inflammation in a mouse model of liver failure [120]. Although these studies suggest that CCR5 antagonists could play a therapeutic role in hepatitis B, the studies in hepatitis C have been conflicting and require further investigation [116,121,122].

## Potential uses

### Transmission

Critical to acquisition of HIV is the chemokine receptor CCR5. The lack of surface expression of CCR5 (homozygosity for CCR5Δ32) is protective against HIV acquisition, and individuals with heterozygous expression of CCR5 have a reduced rate of disease progression [13-15,123]. R5-tropic viruses are almost always the predominant strain in newly infected individuals in both adults and children [124,125], and this data suggests that drugs that block the CCR5 receptor might be particularly effective in the prevention of transmission.

Animal model studies have targeted the CCR5 receptor for prevention of transmission. Analogs of RANTES, a natural ligand of CCR5, have been evaluated as a microbicide and prevented vaginal SHIV transmission in Rhesus macaques [126-128]. Protection against a vaginal SHIV challenge was also provided by vaginally delivered fusion inhibitors and CCR5 antagonists [129,130]. Finally, oral CCR5 antagonists have protected against vaginal SHIV challenge in macaques [131]. These studies demonstrate the feasibility of developing microbicide candidates with CCR5 antagonists, but the challenges of developing a practical and effective formulation/delivery system for humans remain [132]. Further investigation is ongoing with a recent report of incorporating two antiretroviral agents, including maraviroc, into an elastomer vaginal ring [133].

For the reasons noted above, maraviroc seems particularly well suited to be incorporated as a component of regimens for post-exposure (occupational or non-occupational) or pre-exposure prophylaxis, especially because maraviroc attains good levels in both female and male genital secretions and in male rectal tissue [67,70,134]. Trials to evaluate maraviroc as part of PEP are planned [135]. There has been only one reported case of maraviroc as part of occupational PEP, a medical student stuck with a needle from a heavily treatment experienced HIV-infected man [136]. Although it is pregnancy category B, there is no published experience with maraviroc in the prevention of mother to child transmission, but its characteristics merit consideration for preventing vertical transmission [65].

### Intensification

The maraviroc arms in the MOTIVATE trials [75], the non-R5 tropism trial [78], and the MERIT trial [63] all demonstrated CD4 increases significantly greater than the comparator arms. A meta-analysis of clinical trials using CCR5 antagonists also found a significantly greater CD4 increase in CCR5 antagonist arms compared to regimens without one [137]. This finding led to adding maraviroc in virally suppressed patients who have inadequately reconstituted CD4 counts. A number of very small studies have evaluated this question, but none have demonstrated significant increases in CD4 counts [138-140]. Of interest, one of these studies [138] as well as others [141,142] have reported downregulation in immune activation, a finding consistent with data from hepatitis B and C infections and graft versus host disease. This possibility suggests potential benefit beyond the antiviral effects of CCR5 antagonists.

### Organ transplantation

Due to the role of CCR5 receptors in cell recruitment, migration and activation and the finding of lower rejection rates in renal transplant recipients homozygous for CCR5∆32, there has been an interest in modulation of CCR5 in the organ transplant setting [143]. Studies have evaluated both acute and chronic rejection in cardiac allograft transplantation in mice. Several studies evaluated mice that received cardiac allograft transplantation and cyclosporine and demonstrated that the mice that were CCR5 deficient or treated with a CCR5 antibody had prolonged allograft survival [144-146]. Prolonged allograft survival and decreased mononuclear infiltrate in the graft were also seen in mice treated with an anti-CCR5 antibody and Rapamycin [147]. When the CCR5 antagonist, TAK-779, was administered to mice receiving cardiac transplants, reductions in the severity of intimal lesions and the number of graft infiltrating lymphocytes and attenuation of alloantigen-specific T-lymphocyte proliferation and IFN-γ production were seen [148]. Another study used TAK-779 in both a murine cardiac and islet cell transplant model and found that treatment resulted in decreased chemokine, cytokine and chemokine receptor expression, prevented recruitment of lymphocytes into the allografts, and attenuated the development of chronic vasculopathy [149]. Similarly, in the cynomolgus monkey cardiac allograft model, diminished activity and recruitment of CCR5-bearing leukocytes into the graft were attenuated by use of a CCR5 antagonist, though graft survival was only marginally prolonged [150].

In allogeneic bone marrow transplantation, CCR5 has been noted to be a marker for and has been implicated in the pathogenesis of graft versus host disease (GVHD) [151]. The absence of CCR5 has been associated with a lower risk of GVHD [152]. Use of an anti-CCR5 antibody has been shown to reduce GVHD-associated liver injury in a mouse model of GVHD [120]. These studies suggest that CCR5 antagonists may have a role in prolonging graft survival in solid organ and bone marrow transplantation and merit further investigation.

## Other considerations

The CCR5 coreceptor modulates migration and activation of cells expressing the receptor, and so it may play a role in infections other than HIV.

### West Nile Virus (WNV)

In mice WNV infection upregulated CCR5 and its ligand CCL5, which was associated with infiltration of CD4 and CD8 cells into the central nervous system (CNS); additionally, WNV infection in the absence of CCR5 was uniformly fatal [153]. A retrospective study in human cohorts revealed an association between CCR5 deficiency and symptomatic WNV disease, including death [154,155]. However, this association was not seen with heterozygous CCR5∆32. A more recent study in U.S. blood donors concluded that CCR5 deficiency was not by itself a risk factor for WNV acquisition but was associated with clinical symptoms of disease [156]. The clinical implications of these findings for patients treated with CCR5 antagonists are not yet known. Since a CCR5 antagonist is unlikely to block all CCR5 receptors in the body, treated patients may respond to WNV infection in a manner similar to individuals with heterozygous CCR5∆32. Further data from cohort studies and clinical trials are needed before the impact of CCR5 antagonists on WNV infection will be fully elucidated.

### Tick-borne Encephalitis (TBE)

Like WNV, TBE virus is a flavivirus that can be associated with severe CNS disease. The findings with WNV led to evaluations of the role of CCR5 in a small cohort of Lithuanian patients. TBE was more frequent in CCR5∆32 homozygotes and the allele prevalence was higher among TBE patients than TBE-naïve patients with aseptic meningoencephalitis [157]. Further studies in larger populations and in populations treated with CCR5 antagonists are needed to confirm this association and determine the potential impact of treatment with CCR5 antagonists on flavivirus and other infections.

## Future directions

The future roles for CCR5 antagonists in both the prevention and treatment of HIV infection are likely to expand. Recent data demonstrate the importance of CCR5 receptor density for HIV infection [158]. The density of CCR5 defines both the infectability of target PBMC as well as the kinetics of HIV replication in infected cells. Human CD8-depleted PBMC were not able to sustain HIV infection if CCR5 receptor density was less than 2,300 molecules per cell [158]. Physiological levels of CCR5 range between 2,000 molecules to 10,000 molecules per cell and can vary based on the state of immune activation.

HIV entry inhibitors’ potency is directly impacted by the level of CCR5 expression. These include CCR5 antagonists, human antibodies that block viral entry, and fusion inhibitors. Mild differences in CCR5 density are associated with significant differences in IC50 and IC90 of each inhibitor [158-160]. These observations would suggest that as the degree of immune activation increases with progressive HIV-induced immune dysregulation, the *in vivo* potency of the entry inhibitor would be altered. In addition, these data suggest that therapy targeting immune activation may decrease CCR5 density, and thereby enhance the antiviral potency of entry inhibitors.

Our group has recently demonstrated that agents that block the progression of cell cycle at the G1- S interphase, are associated with downregulation of CCR5 expression. This decrease in CCR5 density is associated with an increase in potency of both CCR5 antagonists and HIV fusion inhibitors [160-162]. This enhancement of antiviral activity was to the degree that CCR5-resistant isolates demonstrated IC50’s and IC90’s of wild type isolates when tested in combination with G1 cell cycle agents, or on cells with low level of CCR5 expression [159]. Although speculative, the clinical implication of these findings is that the potency of CCR5 antagonists can be significantly enhanced if used in combination with an agent that causes down-regulation of CCR5.

Current clinical development of CCR5 agents has been approached as if these agents were just an additional antiviral drug to a growing number of effective anti-HIV drugs; but in reality these drugs represent an entire new paradigm in HIV therapeutics. Rather than acting on HIV viral targets, CCR5 agents directly target a distinct host cell pathway, and as such the “rules” are undoubtedly different than those established for agents that target HIV viral enzymes and HIV fusion proteins. For example, when a non-nucleoside reverse transcriptase inhibitor binds reverse transcriptase, it inhibits that molecule alone, yet when a CCR5 inhibitor binds CCR5 it first alters the density available for effective viral binding. Second it signals a down regulation of the cell’s CCR5 molecules giving an effect that lasts for days. As such, there is a significant dichotomy between drug levels and drug effect. Another example would be the difference in resistance threshold. Host targets are much less likely to alter themselves for the benefit of the pathogen’s survival, in distinction from the tendency of viral targets to mutate to escape immune pressure or drug effect. These features alone should alter what we define as the future “rules” for the clinical use of CCR5 agents.

Over the past 25 years significant progress has been made in HIV therapeutics. Over the next 25 years, more advancement will certainly come. One area poised for immediate progress is the broad application of HIV antiretroviral therapy as the centerpiece of 21^st^ century HIV prevention efforts. Fundamental to all transmissible infectious diseases is that the infectivity of those infected drives the epidemic. In addition, application of the knowledge of how a pathogen completes its transmission cycle is crucial. HIV is mainly transmitted across mucous membranes via the transmitting virus R5 tropic stains which enter the new host via CCR5 receptors as long as CCR5 density is adequate. As such, it is clear that CCR5 antagonists will play a major role in the biological prevention of HIV transmission and acquisition. It is anticipated that clinical development pathways will carefully evaluate the role of CCR5 antagonists in the transmission of HIV among discordant couples, both when included as a component of the antiviral therapy of the index case, as well as its role as a chemoprophylactic drug for high risk non-HIV infected partners. Also, of importance is the role CCR5 agents may play in blocking re-infection among individuals who are infected, but at risk for re-infection. The future role of agents for targeting CCR5 is urgently waiting to be fully defined and will require years of focused clinical investigation; yet this first class targeting host cell pathways is poised to herald a new wave in HIV therapeutic and biological HIV prevention.

## Competing interests

BLG, DJR, RRR have no competing interests.

## Authors' contributions

All authors conceived of the manuscript, and participated in its design and coordination. All authors made intellectual contributions and participated in the acquisition, analysis and interpretation of literature data, have been involved in drafting the manuscript, and approved the final manuscript.
